# Differential associations of resting heart rate and heart rate variability indices with cardiovascular and coronary heart disease mortality risk: the prospective KORA study

**DOI:** 10.1038/s41598-026-66516-y

**Published:** 2026-08-18

**Authors:** Kolade Oluwagbemigun, Dan Ziegler, Alexander Strom, Margit Heier, Gidon J. Bönhof, Michael Roden, Wolfgang Rathmann, Christa Meisinger, Annette Peters, Stefanie M. Hauck, Agnese Petrera, Moritz F. Sinner, Stefan Kääb, Barbara Thorand, Christian Herder

**Affiliations:** 1 Institute for Clinical Diabetology, German Diabetes Center, Leibniz Center for Diabetes Research at Heinrich Heine University Düsseldorf, Düsseldorf, Germany; 2 German Center for Diabetes Research (DZD), Partner Düsseldorf, München-Neuherberg, Germany; 3 Institute of Epidemiology, Helmholtz Zentrum München, German Research Center for Environmental Health, Neuherberg, Germany; 4 KORA Study Centre, University Hospital Augsburg, Augsburg, Germany; 5 Department of Endocrinology and Diabetology, Medical Faculty and University Hospital Düsseldorf, Heinrich Heine University Düsseldorf, Düsseldorf, Germany; 6 Institute for Biometrics and Epidemiology, German Diabetes Center, Leibniz Center for Diabetes Research at Heinrich Heine University Düsseldorf, Düsseldorf, Germany; 7 Epidemiology, Medical Faculty, University of Augsburg, Augsburg, Germany; 8 German Center for Diabetes Research (DZD), Partner München-Neuherberg, München- Neuherberg, Germany; 9 Institute for Medical Information Processing, Biometry and Epidemiology (IBE), Faculty of Medicine, Ludwig-Maximilians-Universität (LMU), Pettenkofer School of Public Health, München, Germany; 10 German Centre for Cardiovascular Research (DZHK), Partner Site Munich Heart Alliance, München, Germany; 11 Metabolomics and Proteomics Core, Helmholtz Zentrum München, German Research Center for Environmental Health, Neuherberg, Germany; 12 Department of Cardiology, Ludwig-Maximilians-Universität (LMU) University Hospital, München, Germany

**Keywords:** Resting heart rate, Heart rate variability indices, Cardiovascular disease mortality risk, Coronary heart disease mortality risk, Glucose tolerance status, Normal glucose tolerance, Prediabetes, Type 2 diabetes, Cardiology, Diseases, Endocrinology, Medical research, Risk factors

## Abstract

Limited data exist on the relationship of resting heart rate (RHR) and heart rate variability (HRV) indices with cardiovascular (CVD) and coronary heart disease (CHD) mortality in individuals with and without (pre)diabetes. We investigated overall and glucose tolerance status-specific association of RHR and a large number of HRV indices with incident long-term CVD and CHD mortality. RHR and 66 HRV indices were obtained from five-minute electrocardiogram performed at baseline (KORA S4; 1999–2001) in 1390 adults from the prospective Cooperative Health Research in the Region of Augsburg (KORA). CVD and CHD mortality were ascertained over a 22-year follow-up. Linear and non-linear association of RHR and indices with mortality in the overall sample and normal glucose tolerance (NGT), prediabetes and type 2 diabetes subsamples were determined using Cox models. Independent of covariates, RHR was directly associated with CVD (1.20 [1.04, 1.39], *P* = 0.013) and CHD (1.32 [1.08, 1.62], *P* = 0.007) mortality in the overall study sample. Additionally, very-low frequency power (VLF) was directly associated with CVD mortality (1.17 [1.01, 1.37]; *P* = 0.038) in NGT. Square root of the mean squared differences of successive NN intervals (RMSSD) showed non-linear association with CVD mortality in type 2 diabetes. Number of data points in the 8th column of a 12 × 12 matrix (SPPA_c_8) was inversely associated with CVD mortality (0.54 [0.32, 0.93], *P =* 0.025) in prediabetes. These findings suggest that RHR and HRV indices provide differential and complementary insights into long-term CVD mortality risk in the general population.

**Supplementary Information:**

The online version contains supplementary material available at 10.1038/s41598-026-66516-y.

## Introduction

Cardiovascular diseases (CVD), a group of diseases including coronary heart disease (CHD), are the leading cause of mortality globally^1,2^. Despite novel therapeutic approaches, CVD mortality is still high in some global regions such as central Europe^3^. Resting heart rate (RHR) reflects underlying cardiovascular physiology and is a well-established determinant of cardiac output^4^. In addition to RHR, heart rate variability (HRV), a measure of the fluctuations and variations in time intervals between successive heartbeats, has also become one of the noninvasive cardiovascular biomarkers^5,6^. RHR reflects baseline autonomic tone, while HRV indices provide some insight into the autonomic nervous system-mediated modulation of the heart rate^7^. Despite their relationship, HRV is neither uniformly nor simply a surrogate of RHR^8,9^. HRV indices comprise the traditional linear time- and frequency-domain indices and novel non-linear indices^10^. Therefore, RHR and HRV indices might provide differential and complementary insights into CVD and CHD mortality risk.

A recent systematic review of meta-analyses and original epidemiological studies concluded that higher RHR is associated with higher CVD mortality risk^4^. However, studies that are more recent have yielded heterogeneous findings; such that time-updated RHR, but not baseline RHR was associated with CVD mortality risk^11^, whereas in another study no association was found between RHR and CVD mortality risk^12^. Further studies showed a non-linear association of RHR with CVD mortality risk^13^ or a linear association of RHR with CVD mortality risk^14^. In addition, evidence from recent decades suggests links between HRV indices and CVD mortality risk^15–21^. Standard deviation of all normal-to-normal (NN) intervals (SDNN), standard deviation of the averages of N-N intervals (SDANN), square root of the mean square of differences between adjacent N-N intervals (RMSSD), percentage of the number of pairs of adjacent N-N intervals differing by more than 50 ms (pNN50), very-low frequency power (VLF), low frequency power (LF), high frequency power (HF) and total power (TP) were the indices most commonly investigated for association with CVD mortality risk^15,16^. Overall, lower values of SDNN^15,16^, VLF^16^, LF^15,16^, HF^16^ and TP^16^ were independently associated with higher CVD mortality risk, which was in contrast to lacking associations for SDANN, RMSSD and pNN50^15^. However, there is a dearth of studies linking these common indices to CHD mortality risk. Additionally, the few studies on non-linear indices include pooled analysis that showed independent associations of prolonged QT-interval length with higher CVD and CHD mortality risk^17^ as well as original studies showing independent associations of higher QT variability index^18^, steep slope of the power-law regression line^19^ and lower short-term fractal scaling exponent^20^ with higher CVD mortality risk.

There is an intricate interplay between the occurrence of CVD and type 2 diabetes (T2D)^21^. This resonates with high fasting plasma glucose being among the important risk factors of global CVD mortality^1^ and both prediabetes and T2D have been linked to CVD mortality^22–24^.Although a causal relationship between HRV and T2D seems unlikely^25^, individuals with T2D generally have lower HRV indices when compared to those with normal glucose tolerance (NGT)^26,27^. A few studies have demonstrated that higher RHR is associated with increased CVD mortality risk in individuals with T2D^28–30^. The limited number of studies that have examined the association of similar HRV indices in relation to CVD mortality risk in individuals with T2D yielded inconsistent results^31–34^. Furthermore, prediabetes, a heterogeneous metabolic disorder that bridges NGT and T2D, is associated with adverse cardiometabolic outcomes^35^. However, the association of RHR and HRV indices with either CVD or CHD mortality risk in individuals with prediabetes is underreported. Importantly, only few studies^12,31^ exist on the association of either RHR or HRV indices with long-term (up to 20-year) CVD and CHD mortality risk. Importantly, there is a paucity of data on the association between a large number of HRV indices and mortality.

Therefore, the present prospective epidemiological study aims to examine the overall as well as NGT-, prediabetes- and T2D-specific association of RHR and a large number of HRV indices with incident long-term CVD and CHD mortality risk.

## Methods

The data are subject to national data protection laws. Therefore, data cannot be made freely available in a public repository. However, data can be requested through an individual project agreement with KORA. To obtain permission to use KORA data under the terms of a project agreement, please use the digital tool KORA.PASST (https://epi.helmholtz-muenchen.de/).

### Study population and design

The current study is based on data from the population-based Cooperative Health Research in the Region of Augsburg (KORA) S4 cohort (1999–2001, baseline) with up to 22 years of follow-up. In 1999–2001, study participants were recruited from the region of Augsburg (Germany) using random sampling and random selection of 16 towns and villages from 70 communities. Each community underwent sex- and age-stratified sampling, with four of the strata comprising men and women aged 55 to 74 years. Medical history was obtained through a structured interview, and various medical assessments such as electrocardiogram recordings were performed. Details of the design of the KORA cohort and assessments have been thoroughly described elsewhere^36–39^. All investigations were conducted in accordance with the Declaration of Helsinki and all participants provided written informed consent. The ethics committee of the Bavarian Chamber of Physicians, Munich approved all study protocols.

**Fig. 1:**
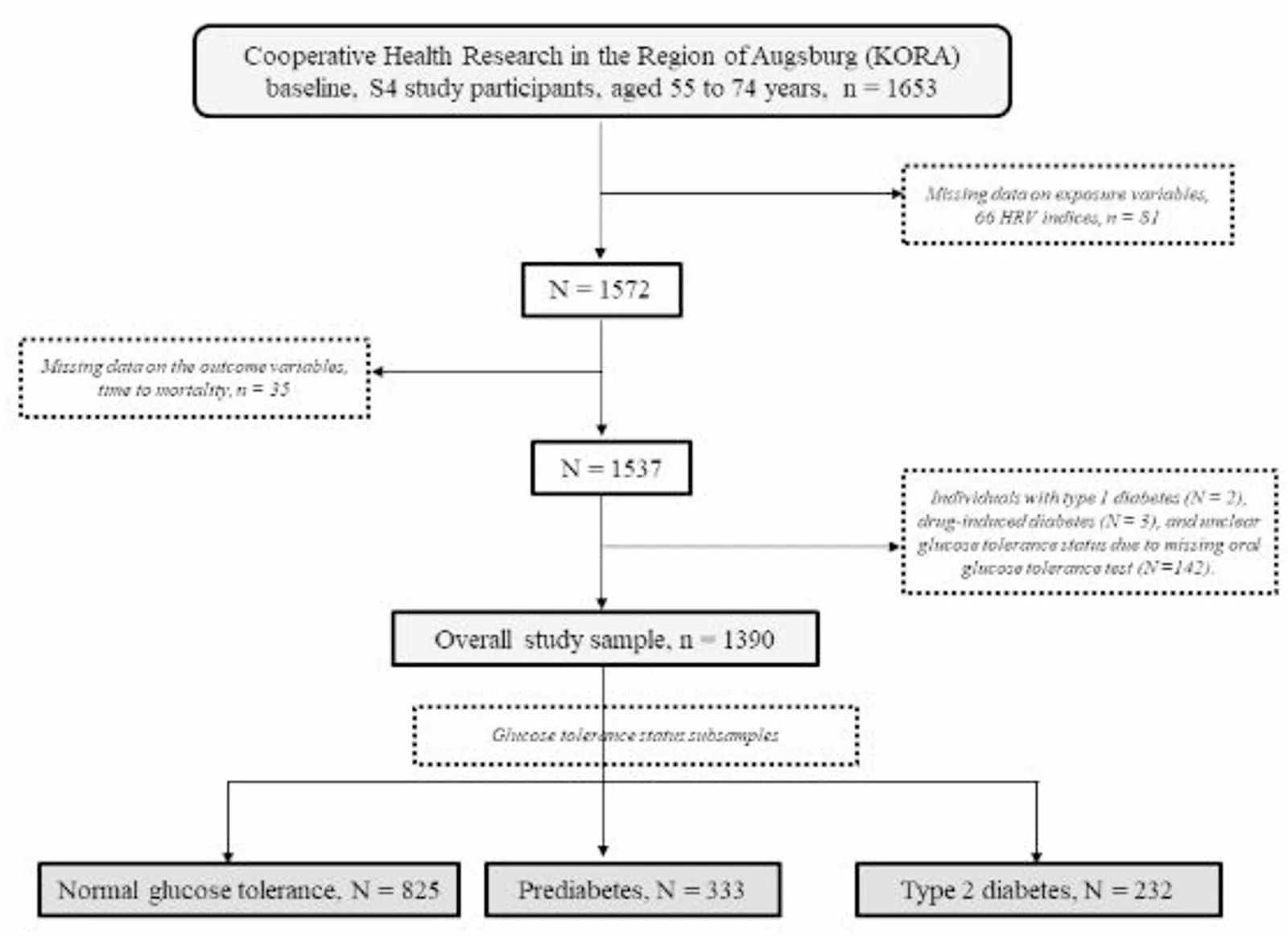
Flow chart of the study population.

The present analysis is based on 1653 individuals at KORA S4 (baseline) aged 55 to 74 years. We sequentially excluded 81 individuals who had missing data on any of the current exposure variables, i.e. 66 selected HRV indices; 35 individuals with missing data on any of the outcome variables, i.e. time-to-CVD mortality, and individuals with type 1 diabetes (*N* = 2), drug-induced diabetes (*N* = 3), and unclear glucose tolerance status due to missing oral glucose tolerance test (OGTT) (*N* = 142). This resulted in an overall sample of 1390 eligible individuals. Figure 1 depicts the flow chart of the study sample.

### Assessment of the exposure variables: RHR and HRV indices

Electrocardiogram (ECG) was recorded in a standardized manner after 10 min of rest in a supine position in an attenuated atmosphere environment at the study center. ECG electrodes were applied by specifically trained technicians using a DAL square for positioning. 10-second recordings were started once the recording quality was evaluated and noise and artefact optimization were applied. The actual 5-minute recordings started immediately after the 10-second recordings. ECG recordings took place during the working hours of the study center, i.e. not only at one predefined time point. Participants were not fasting nor required to adhere to a specific diet prior to the recording. Time series of the heart rate (tachograms) were extracted from the recordings. Ectopic beats and artefacts were detected and replaced by interpolated ‘normal’ beats by applying an adaptive filter to generate N-N interval time series. Linear and non-linear HRV analysis methods were applied to the filtered tachograms. Further details of the method are provided elsewhere^37,40^. Individuals with atrial fibrillation or flutter, left and right bundle-branch block, second‐ and third‐degree atrioventricular block or sinoatrial block, multiple supraventricular or ventricular extrasystoles, pacemaker therapy, and treatment with class I antiarrhythmics were excluded. We selected a comprehensive set of 66 HRV indices, comprising 17 linear time- and frequency-domain indices and 49 indices from non-linear methods such as symbolic dynamics, detrended fluctuation analysis, compression entropy, traditional and segmented Poincaré plot analysis. The description of the 66 HRV indices is provided in Table S1. RHR (beats per minute, bpm) was calculated as 60,000 divided by the mean N-N interval.

### Assessment of CVD mortality and CHD mortality

KORA study participants were followed up for all-cause and cause-specific mortality such as CVD mortality and CHD mortality by regularly checking the vital status of participants through the population registries inside and outside the study area. Causes of death reported in death certificates were recorded according to the 9th edition of the International Statistical Classification of Diseases, Injuries and Causes of Death (ICD-9). CVD mortality consists of death from diseases of the circulatory system (ICD-9 code 390–459), of which CHD comprised ICD-9 codes 410–414 and sudden death with unknown cause (ICD-9 code 798). CHD mortality also included ICD-9 code 798^41^. The follow-up for death in this current analysis was until the end of 2021. Participants who were not followed up until death were considered event-free (alive) until the date of last contact and their subsequent follow-up was censored. The follow-up time was up to 22 years.

### Assessment of covariates: sociodemographic, anthropometric and lifestyle factors and other biomarkers

Information on age, sex, educational attainment, smoking habits, alcohol consumption, physical activity and medical history were collected in computer-assisted standardized personal interviews conducted by experienced medical staff. Educational attainment was recorded as completed years of schooling. Height, weight, waist circumference, systolic (SBP) and diastolic blood pressure (DBP) were measured at the study visit using standard methods at baseline. Body mass index (BMI, kg/m^2^) was calculated from weight and height. Smoking habits and alcohol consumption were self-reported. Smoking status was categorized as non-smokers, former smokers, and current (regular and irregular) smokers. Alcohol consumption was based on reported intake of beer, wine, and liquor on one weekday and the weekend and expressed in grams/day. Participants estimated the duration and frequency of their weekly exercise across summer or winter. They were categorized as either physically active (one hour or more sports/week) or inactive. Blood pressure was measured using an OMRON HEM-705CP (Omron Corporation, Tokyo, Japan) three times at the right arm after a 5-minute resting period. The mean of the second and third measurements was used for analysis. Medication in the last 7 days was recorded on the basis of the packaging brought to the study visit. Coding was done using the Anatomical Therapeutic Chemical Classification System codes. Detailed information on the assessment of these covariates are available elsewhere^42–45^. From plasma samples, high-density lipoprotein cholesterol (HDL-cholesterol), low-density lipoprotein cholesterol (LDL-cholesterol) and triglycerides were measured by enzymatic methods^46^. Glycated hemoglobin A1c (HbA1c) was measured by immune turbidimetric assays^47^, while high-sensitivity C-reactive protein (hsCRP) was quantified using a high-sensitivity latex-enhanced nephelometric assay on a BN II System analyser (Dade Behring, Marburg, Germany)^48^. OGTT was performed using standard procedure on those without previously known diabetes. Individuals were categorized into NGT, prediabetes (isolated impaired fasting glucose, isolated impaired glucose tolerance, combined impaired fasting glucose/impaired glucose tolerance) and T2D (newly detected T2D from OGTT and previously known T2D) in accordance with the criteria of the American Diabetes Association^49^. All covariates were assessed at baseline.

### Statistical analysis

#### Descriptive analysis

Continuous and categorical baseline characteristics (covariates) of the overall study sample were summarized as medians (25% and 75% percentiles) and counts (percentages), respectively. Continuous and categorical covariates stratified by NGT, prediabetes and T2D were compared with the Kruskal–Wallis rank-sum test and Pearson’s Chi-squared test, respectively.

### Modeling the associations of RHR and HRV indices with CVD and CHD mortality risk

Firstly, we determined the association of RHR as a continuous variable with CVD and CHD mortality by fitting semiparametric proportional hazards (PH) regression (Cox) model on the overall sample (*N* = 1390) and NGT (*N* = 825), prediabetes (*N* = 333) and T2D (*N* = 232) subsamples. Time-to-CVD and time-to-CHD mortality were modeled as the number of days from the individual’s study enrolment until either the occurrence of the event (death) or the last time they were known to be alive. We fitted crude and covariate-adjusted Cox models. We used directed acyclic graphs to select of a sufficient set of covariate (Figure S1). These covariates were age (years), sex (women and men [reference]), BMI (kg/m^2^), waist circumference (cm), SBP (mmHg), DBP (mmHg), HDL-c (mmol/L), LDL-c (mmol/L), triglycerides (mmol/L), educational attainment (years), physical inactivity (inactive and active [reference]), smoking status (smokers, ex-smokers, and non-smokers [reference]), alcohol consumption (g/day), prevalent myocardial infarction, each medication usage (angiotensin antagonists, angiotensin-converting enzyme inhibitors, calcium antagonists, beta blockers, statins, non-steroidal anti-inflammatory drugs (users and non-users [reference]), hsCRP (mg/L) and HbA1c (mmol/mol). Hence we examined a crude model and a model comprising all these aforementioned covariates (full covariate-adjusted model). In addition, penalized smoothing splines were used to assess potential non-linear relationships of RHR with mortality risks in full covariate-adjusted models. The optimal smoothing was based on model-specific Akaike Information Criterion with zero degrees of freedom. To validate non-linearity, we compared the performance of the spline model and continuous model by computing the Bayes factor. A Bayes factor greater than one^50^ was considered as evidence in favor of the spline model and vice versa. We considered a non-linear association to be convincing if the non-linear RHR term was statistically significant and the spline model was favored over the continuous model (Bayes factor > 1).

Secondly, we determined the association of the common HRV indices (SDNN, SDANN, RMSSD, pNN50, VLF, LF, HF and TP). The methodology, in terms of examined study samples, covariates, outcomes, as well as continuous and spline modeling and comparison, was similar to that described above.

Finally, we explored the association of 66 HRV indices (Table S1), which included the common HRV indices, with CVD or CHD mortality. Since indices are conceptually related, they were jointly modelled in a training-testing split validation approach in order to recover robust and non-redundant indices while accounting for interactions among them. A 20% of the data was held out (model fitting dataset) and we fitted penalized Cox models by component-wise likelihood-based boosting to the remaining 80% (variable selection dataset). The penalized Cox models selected a subset of predictor variables (all 66 indices and aforementioned covariates), which together are strongly related with mortality while penalizing their regression coefficients to minimize overfitting. Models were fitted with optimal number of boosting steps and penalty parameters were obtained by 10-fold cross-validation. The resulting non-zero estimate predictor variables were validated on the model fitting dataset using standard Cox models.

Missing values of covariates were single-value imputed using the non-parametric multivariate imputation by the chained random forest. RHR, HRV indices and continuous covariates were centered and scaled. Hence, hazard ratios (HR) for continuously modeled RHR and HRV indices indicated change in mortality risk per one-SD increase. We checked the consistency of our models with the PH assumption for each variable separately as well as globally. Only a few models fully satisfied the PH assumption. We estimated robust standard errors to account for any lack of PH as well as omitted covariates and any improper functional form for continuous predictor variables^51^. Reliable associations for analyses of RHR and common indices were statistically significant estimates in both crude and full covariate-adjusted models, while for the analyses of 66 indices are validated estimates, that is, statistically significant in the model fitting datasets.

### Bias analysis

If RHR or common indices showed statistically significant associations in the crude model, we assessed the impact of follow-up time by comparing HR at early (less than five years) and late (five and more years) follow-up. We checked for reverse causation, in which RHR or HRV indices could have been substantially altered among individuals closer to dying, by excluding participants with less than five years of follow-up. This is equivalent to comparing HR of late follow-up with HR of the entire follow-up. Further, we assessed the robustness of the results in a subset of the overall study sample who had indices additionally assessed 14 years later. We estimated the association of baseline, follow-up and change in RHR and HRV indices with CVD and CHD mortality risk. The association of SDNN and RMSSD with CVD mortality seems to vary by sex^31^, hence we checked for potential effect modification fitting interactions between these indices and sex.

### Supplementary analysis

We modeled dichotomized indices (below the fifth percentile of individuals with NGT vs. fifth percentile or more of NGT as reference, similar to a previous study^37^. In addition, we explored the association of RHR (continuous and spline) and HRV indices (continuous, spline and dichotomized) with all-cause mortality risk. Finally, we determined bivariable associations of RHR and HRV indices with selected covariates in the overall study sample.

### Statistical power

We estimated the statistical power of the four study samples using pooled covariate-adjusted hazard ratio (HR) of 1.51 (relative risk of 1.33) for high RHR (versus low RHR as reference)^52^ and HR of 1.52 for low SDNN (versus high SDNN as reference) with CVD mortality risk^15^, each sample size, their number of CVD and CHD deaths and *P* of 0.05. For RHR, the statistical powers for CVD and CHD mortality risk were 92% and 64%, respectively in the overall sample; 67% and 33% respectively in NGT subsample; 34% and 21% respectively in prediabetes subsample and 44% and 26% respectively in T2D subsample. For SDNN, they were 93% and 65%, respectively in the overall sample; 68% and 34% respectively in the NGT subsample; 35% and 22% respectively in the prediabetes subsample and 45% and 26% respectively in the T2D subsample.

All statistical analyses were performed using R version 4.3.3. Statistical significance was set at *P* < 0.05.

## Results

### Descriptive analysis

Table 1 provides an overview of the basic characteristics of the overall study population (*N* = 1390). The median age and BMI were 64 years and 28 kg/m^2^, respectively. Among them, 52% were men. After a median follow-up of 21 years, there were 268 CVD and 127 CHD deaths. A majority (825) of the study population were individuals with NGT, while 333 (24%) and 232 (17%) individuals had prediabetes and T2D, respectively. There were 135 CVD and 55 CHD deaths in NGT, 57 CVD and 32 CHD deaths in prediabetes, 76 CVD and 40 CHD deaths in T2D. Notably, all basic characteristics, except smoking status, myocardial infarction, use of angiotensin antagonists, and non-steroidal anti-inflammatory drugs, were associated with glucose tolerance status (Table 1). The median RHR and SDNN in the overall study population were 68 bpm and 27 ms, respectively. RHR and 25 indices were associated with glucose tolerance status (Table S2).

**Table 1:** Basic characteristics of the study population.

	Overall (*N* = 1390)	Normal glucose tolerance (*N* = 825)	Prediabetes (*N* = 333)	Type 2 diabetes (*N* = 232)	*P*
Age, years	64 (59, 69)	63 (59, 68)	65 (61, 69)	65 (61, 70)	< 0.001
Women, *n* (%)	669 (48.1%)	437 (53.0%)	135 (40.5%)	97 (41.8%)	< 0.001
Body mass index, kg/m²	28 (26, 31)	27 (25, 30)	29 (27, 32)	30 (28, 34)	< 0.001
Waist circumference, cm	96 (89, 103)	93 (85, 100)	99 (93, 106)	101 (96, 110)	< 0.001
Educational attainment, years	10 (10, 12)	10 (10, 12)	10 (10, 12)	10 (8, 11)	0.003
Alcohol consumption, g/day	7 (0, 23)	6 (0, 23)	14 (0, 28)	5 (0, 22)	0.011
Smokers, *n* (%)	190 (13.7%)	124 (15.0%)	36 (10.8%)	30 (12.9%)	0.054
Physically inactive	798 (57.6%)	429 (52.3%)	208 (62.7%)	161 (69.4%)	< 0.001
Systolic blood pressure, mmHg	135 (123, 148)	130 (118, 144)	140 (126, 152)	145 (133, 158)	< 0.001
Diastolic blood pressure, mmHg	80 (73, 87)	78 (72, 86)	82 (75, 89)	83 (76, 89)	< 0.001
High-density lipoprotein cholesterol, mmol/L	1.4 (1.2, 1.7)	1.5 (1.3, 1.8)	1.4 (1.2, 1.7)	1.3 (1.0, 1.5)	< 0.001
Low-density lipoprotein cholesterol, mmol/L	3.9 (3.3, 4.6)	3.9 (3.3, 4.7)	4.1 (3.3, 4.7)	3.7 (3.1, 4.4)	0.003
Triglycerides, mmol/L	1.4 (1.0, 1.9)	1.2 (0.9, 1.7)	1.4 (1.1, 2.1)	1.9 (1.4, 2.9)	< 0.001
High-sensitivity C-reactive protein, mg/L	1.8 (0.9, 3.6)	1.4 (0.7, 3.0)	2.2 (1.2, 4.2)	2.4 (1.1, 5.6)	< 0.001
HbA1c, mmol/mol	38 (36, 41)	38 (34, 40)	38 (36, 41)	45.5 (41, 55)	< 0.001
HbA1c, %	5.6 (5.4, 5.9)	5.6 (5.3, 5.8)	5.6 (5.4, 5.9)	6.4 (5.9, 7.2)	< 0.001
Myocardial infarction, *n* (%)	61 (4.4%)	29 (3.5%)	16 (4.8%)	16 (6.9%)	0.079
Use of angiotensin antagonists	46 (3.3%)	21 (2.6%)	14 (4.2%)	11 (4.7%)	0.15
Use of angiotensin-converting enzyme inhibitors, *n* (%)	177 (12.8%)	71 (8.6%)	44 (13.2%)	62 (26.7%)	< 0.001
Use of calcium antagonists, *n* (%)	146 (10.5%)	55 (6.7%)	42 (12.6%)	49 (21.1%)	< 0.001
Use of beta blockers, *n* (%)	298 (21.5%)	134 (16.3%)	91 (27.3%)	73 (31.5%)	< 0.001
Use of statins, *n* (%)	140 (10.1%)	73 (8.9%)	32 (9.6%)	35 (15.1%)	0.02
Use of non-steroidal anti-inflammatory drugs, *n* (%)	99 (7.1%)	62 (7.5%)	22 (6.6%)	15 (6.5%)	0.781
CVD mortality, *n* (%)	268 (19.3%)	135 (16.4%)	57 (17.1%)	76 (32.8%)	< 0.001
CHD mortality, *n* (%)	127 (9.1%)	55 (6.7%)	32 (9.6%)	40 (17.2%)	< 0.001
Follow-up time, years	21 (15, 21)	21 (16, 22)	21 (14, 21)	16 (10, 21)	< 0.001

Continuous and categorical covariates are presented as medians (25% and 75% percentiles) and counts (percentages), respectively. *P* is from Kruskal–Wallis rank-sum test or Pearson’s Chi-squared test. *P*, p-value; N, sample size; CVD, cardiovascular disease; CHD, coronary heart disease; type 2 diabetes comprises previously clinically diagnosed type 2 diabetes and newly identified type 2 diabetes based on OGTT.

### Modeling of the association of RHR and HRV indices with CVD and CHD mortality risk

**Fig. 2:**
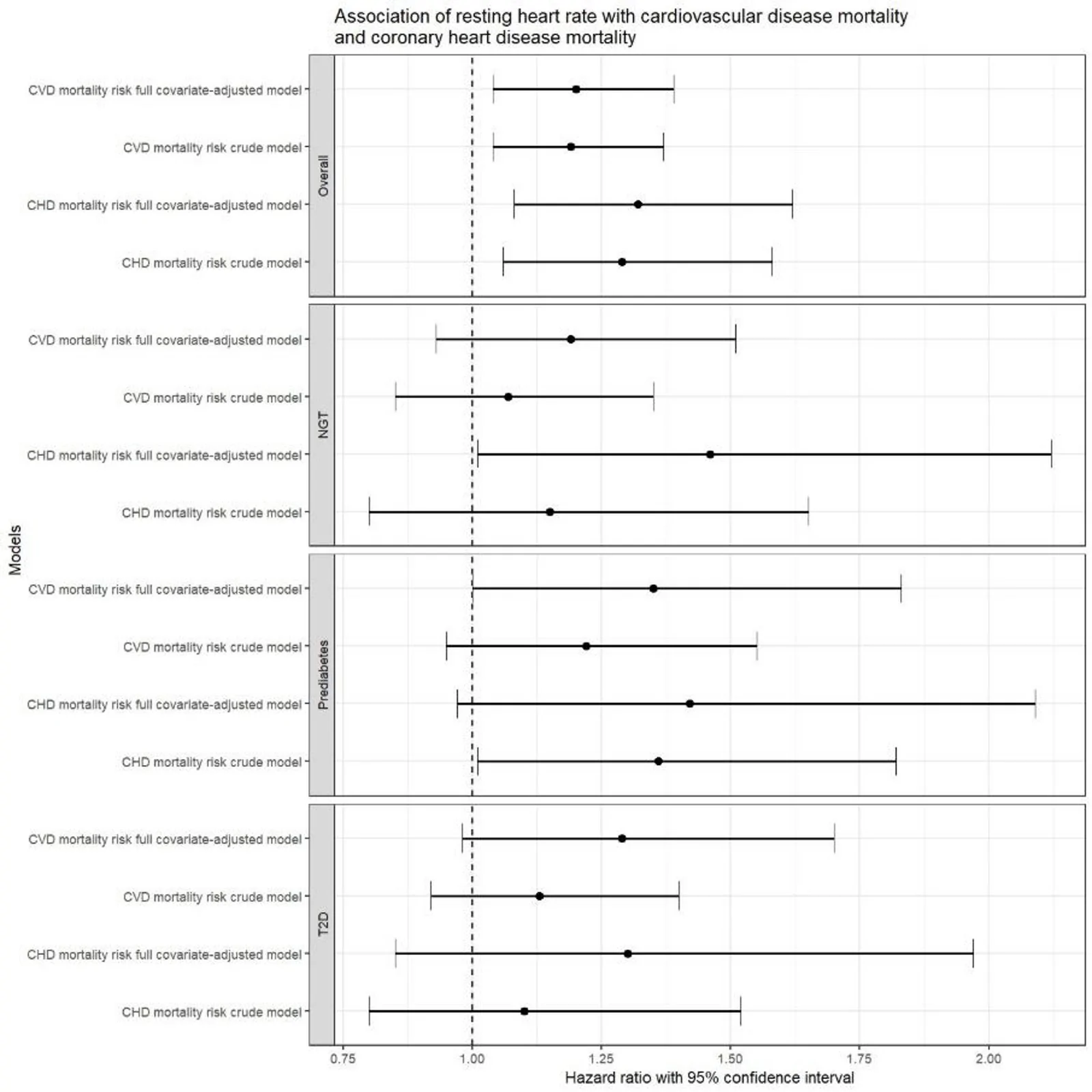
Association of resting heart rate with cardiovascular disease mortality and coronary heart disease mortality in the overall study sample, normal glucose tolerance, prediabetes and type 2 diabetes subsamples.

Figure 2 shows that higher RHR was associated with higher CVD and CHD mortality in the crude and full covariate-adjusted models (CVD: HR 1.20 [1.04, 1.39], *P* = 0.013; CHD: HR 1.32 [1.08, 1.62], *P* = 0.007) in the overall study sample. There was no significant association of RHR with either CVD or CHD mortality risk in the subsamples despite mostly similar effect sizes as in the overall study sample.

**Fig. 3:**
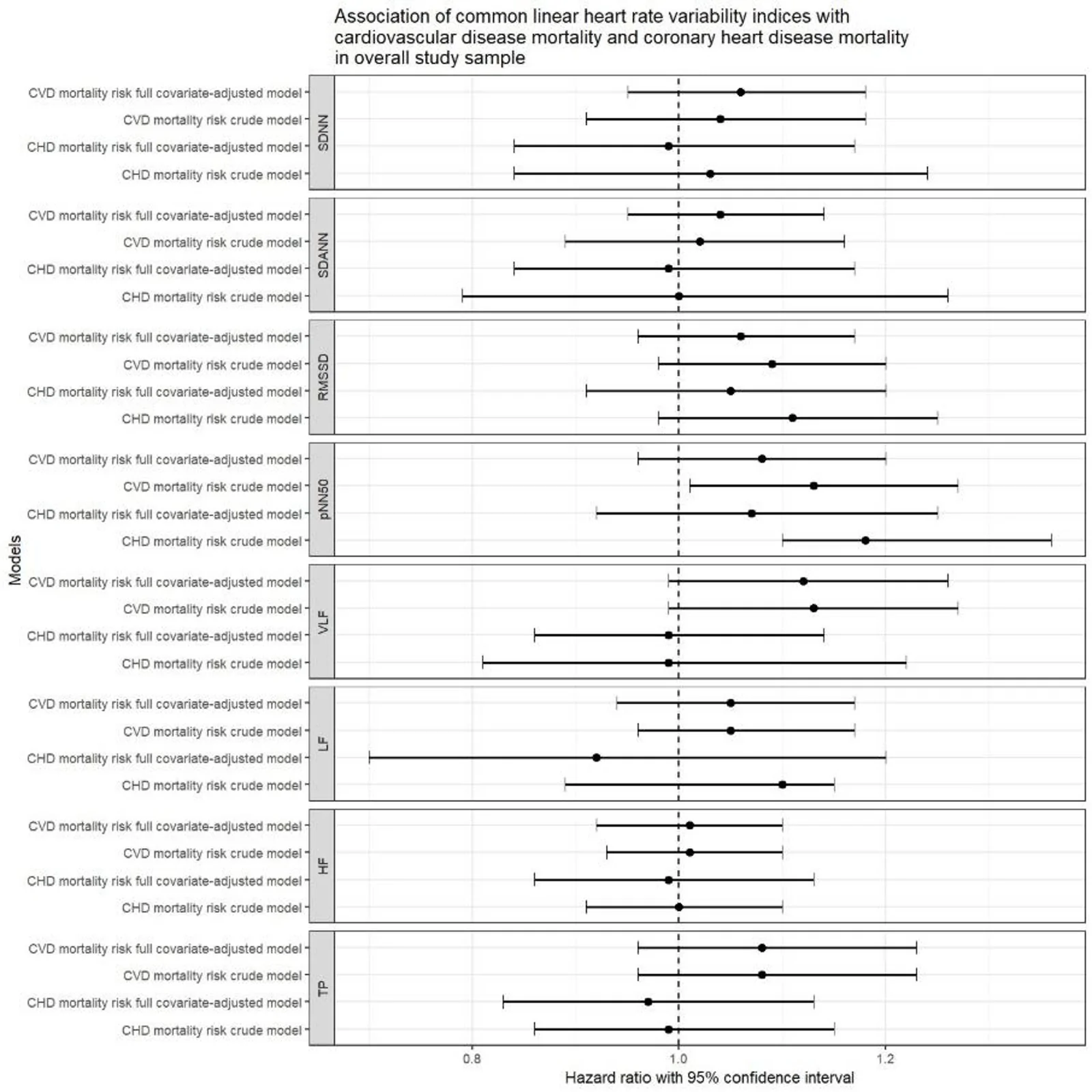
Association of common heart rate variability indices with cardiovascular disease mortality and coronary heart disease mortality in the overall study sample.

**Fig. 4:**
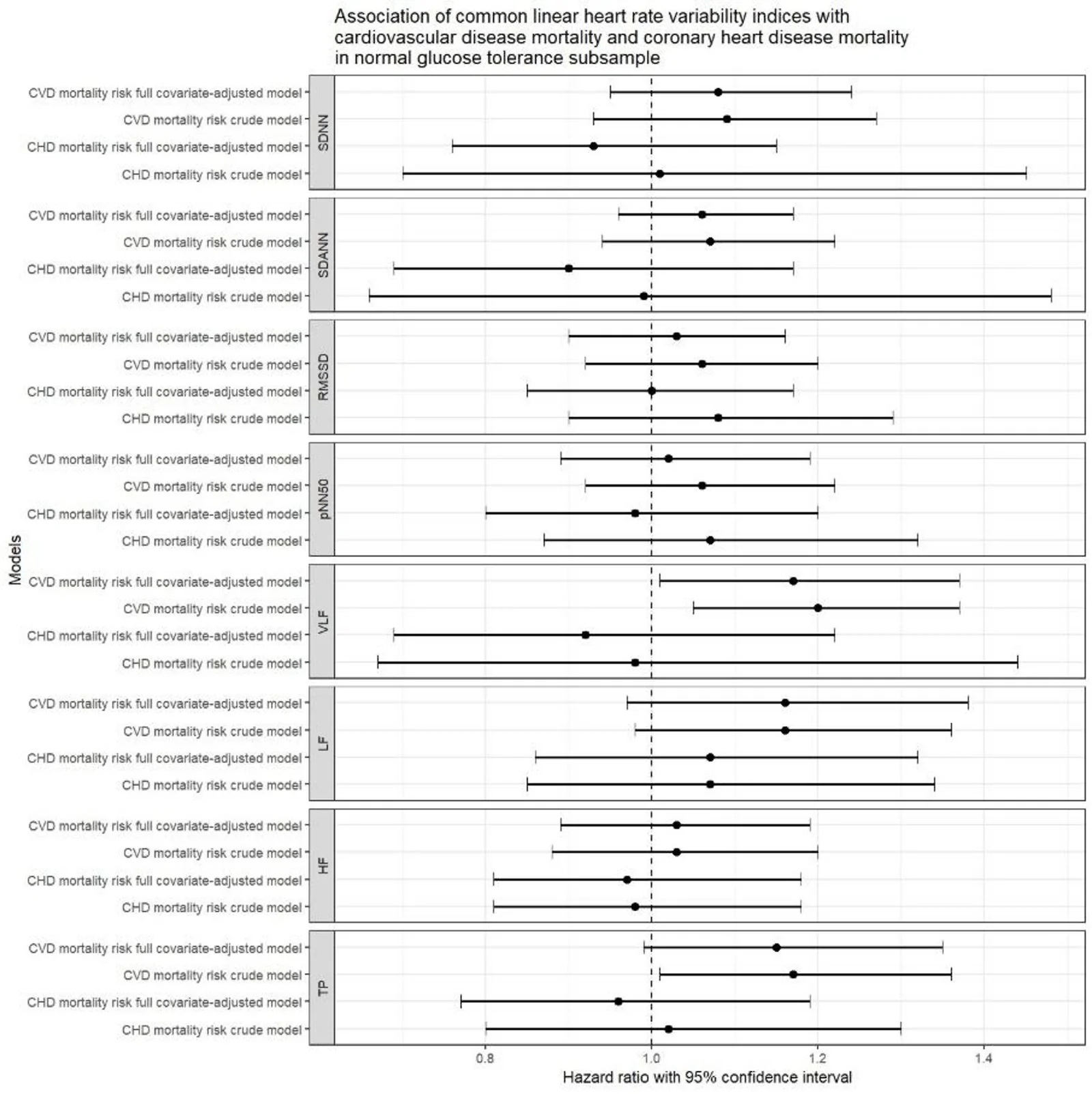
Association of common heart rate variability indices with cardiovascular disease mortality and coronary heart disease mortality in the normal glucose tolerance subsample.

**Fig. 5:**
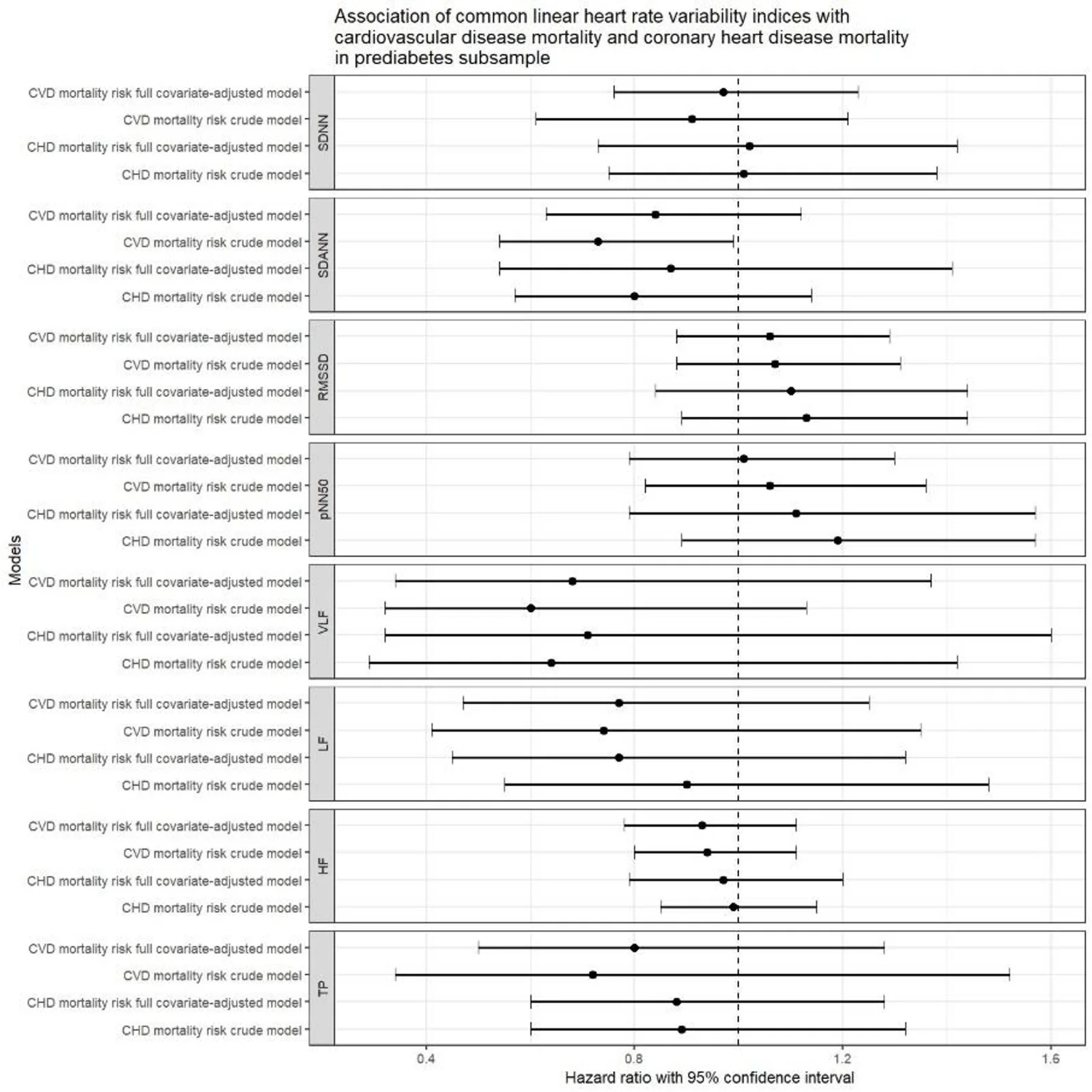
Association of common heart rate variability indices with cardiovascular disease mortality and coronary heart disease mortality in the prediabetes subsample.

**Fig. 6:**
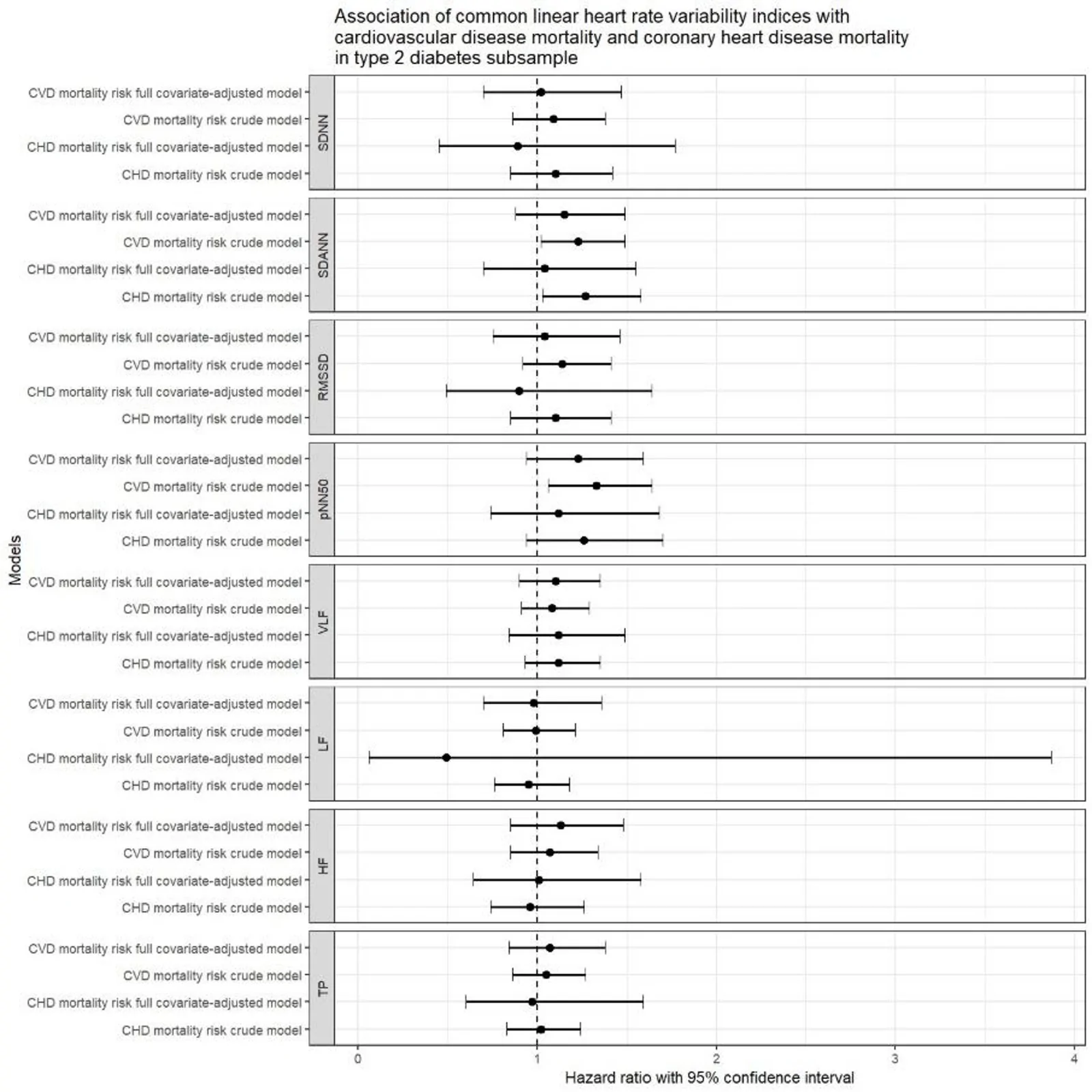
Association of common heart rate variability indices with cardiovascular disease mortality and coronary heart disease mortality in the type 2 diabetes subsample.

Figures 3, 4, 5 and 6 displays the results of the common indices. In the crude model, we observed direct associations of pNN50 with CVD and CHD mortality in the overall sample (Fig. 3), VLF and TP with CVD mortality in the NGT subsample (Fig. 4), SDANN and pNN50 with CVD mortality in the T2D subsample (Fig. 6) and SDANN with CHD mortality in the T2D subsample (Fig. 6). There was an inverse association of SDANN with CVD mortality in the prediabetes subsample (Fig. 5). Only the association of VLF with CVD mortality risk in the NGT subsample remained in the full covariate-adjusted model (Fig. 4) (HR: 1.17 [1.01, 1.37]; *P* = 0.038).

**Fig. 7:**
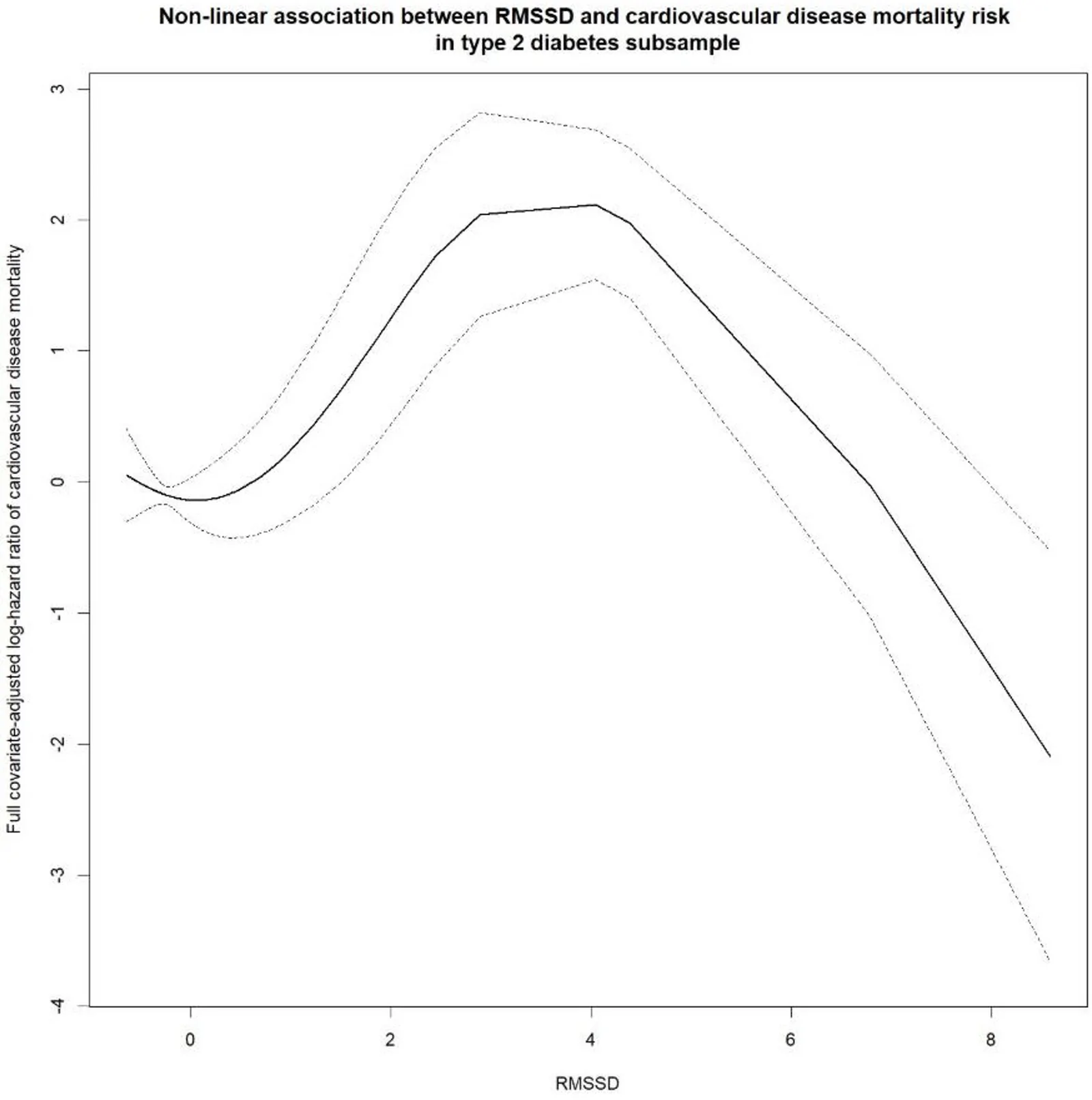
Non-linear association between RMMSD and cardiovascular disease mortality in the type 2 diabetes subsample.

The statistical significance of non-linear terms in spline models and Bayes factor comparisons of continuous and spline models for associations of RHR and HRV indices with CVD and CHD mortality risk are presented in Tables S3 and S4, respectively. The plots of RHR and the indices in the overall sample and subsamples are displayed in Figures S2–S10. While non-linear terms of RHR and most indices were statistically significant (*P* < 0.05), Bayes factors were generally less than one indicating that continuous models generally outperform the spline models. The only exception was the non-linear association between RMSSD and CVD mortality risk in the T2D subsample (*P*_non−linear_ = < 0.001; Bayes factor = 4.02, Table S4). As shown in Fig. 7, there was an S-shaped relationship between RMSSD and CVD mortality risk. Around the mean of RMSSD, a steady decrease in CVD mortality risk was seen with increasing RMSSD. Between 1.5-SD (75 ms) to 5.5-SD (208 ms), increasing RMMSD was associated with increasing risk. Beyond 208 ms, an increasing value was associated with decreasing risk.

Tables 2 and 3 show the subsets of predictor variables associated with CVD and CHD mortality in the variable selection datasets and their validation in the model fitting datasets. In the overall sample, six indices including VLF and Shannon entropy of all data points (SPPA_entropy) were associated with CVD mortality, while two indices including SPPA_entropy were associated with CHD mortality (Table 2). In the NGT subsample, no index was associated with either CVD or CHD mortality risk (Table 3). In the prediabetes subsample, four indices including number of data points in the 8th column of a 12 × 12 matrix (SPPA_c_8) were associated with CVD mortality, while three indices including number of data points in the 8th row of a 12 × 12 matrix (SPPA_r_8) were associated with CHD mortality (Table 3). In the T2D subsample, no index was associated with CVD mortality risk, while two indices including slope of the auto-transformation function (ati) from the maximum of ati (tau = 0) to the subsequent value (tau = 2) (a31rr) were associated with CHD mortality (Table 3). Only the association of SPPA_c_8 with CVD mortality risk in the prediabetes subsample was validated (adjusted HR: 0.54 [0.32, 0.93], *P =* 0.025) (Table 3).

**Table 2:** Association of selected predictor variables, heart rate variability indices and covariates with cardiovascular mortality and coronary heart disease mortality in the overall study sample.

Variable selection dataset (*N* = 1112; *n* = 220^a^, 100^b^)	Model fitting dataset (*N* = 278; *n* = 48^a^, 27^b^)	
CVD mortality^a^		
	*HR (95% CI)*	*P*
ST_2LV	1.43 (0.65, 3.13)	0.374
ASC	0.47 (0.19, 1.16)	0.101
VLF	0.89 (0.72, 1.09)	0.261
SPPA_r_8	0.91 (0.60, 1.38)	0.652
SPPA_entropy	0.89 (0.66, 1.20)	0.436
VALLEY	1.04 (0.77, 1.39)	0.818
Age	2.53 (1.78, 3.60)	< 0.001
Women (ref.: men)	0.63 (0.32, 1.28)	0.201
Waist circumference	1.44 (1.01, 2.05)	0.042
Educational attainment	0.86 (0.62, 1.20)	0.385
Physically inactive (ref.: active)	1.45 (0.69, 3.06)	0.325
Systolic blood pressure	0.94 (0.66, 1.33)	0.712
HbA1c	1.45 (1.06, 1.99)	0.020
Angiotensin-converting enzyme inhibitors non-users (ref. users)	1.67 (0.66, 4.20)	0.278
Smokers (ref. non-smokers)	1.58 (0.58, 4.35)	0.374
CHD mortality^b^		
ST_2LV	0.75 (0.50, 1.12)	0.162
SPPA_entropy	0.83 (0.61, 1.12)	0.219
Age	2.67 (1.81, 3.93)	< 0.001
Women (ref.: men)	0.28 (0.10, 0.78)	0.015
Waist circumference	1.29 (0.75, 2.22)	0.362
Educational attainment	0.83 (0.56, 1.22)	0.342
Angiotensin-converting enzyme inhibitors non-users (ref. users)	1.08 (0.44, 2.68)	0.868

ST_2LV, portion of words of the pattern family “2LV” (the three symbols formed an ascending or descending ramp (e.g. 1 2 4 or 5 4 3); ASC, portion of words of the pattern family “ascending” (the three successive symbols describe a ascending ramp (e.g. 2 3 4 or 0 1 2); SPPA_r_8, number of data points in the 8th row of a 12 × 12 matrix; SPPA_entropy, Shannon entropy of all data points; VALLEY, portion of words of the pattern family “valley” (the second symbol is smaller than the remaining ones forming a valley (e.g. 2 1 2 or 3 1 2); HR, hazard ratio; CI, confidence interval; *P*, p-value; N, sample size; n, number of events; CVD, cardiovascular disease; CHD, coronary heart disease; HR is per one-SD increase for continuous variables; *adjusted HR.

**Table 3:** Association of selected predictor variables, heart rate variability indices and covariates with cardiovascular disease mortality and coronary heart disease mortality in the normal glucose tolerance, prediabetes and type 2 diabetes subsamples.

Variable selection dataset (*N* = 660; *n* = 107^a^, 45^b^)	Model fitting dataset (*N* = 165; 28^a^, 10^b^)	
Normal glucose tolerance subsample		
CVD mortality^a^		
	HR (95% CI)	*P*
Age	2.15 (1.52, 3.05)	< 0.001
CHD mortality^b^		
Age	1.85 (1.19, 2.87)	0.006
Prediabetes subsample		
Variable selection dataset (*N* = 266; *n* = 46^a^, 26^b^)	Model fitting dataset (*N* = 67; *n* = 11^a^, 6^b^)	
CVD mortality^a^		
	*HR (95% CI)*	*P*
amax21rrcor	0.77 (0.25, 2.38)	0.655
QTVInu	0.93 (0.59, 1.47)	0.763
SPPA_c_8	0.54 (0.32, 0.93)	0.025
SPPA_r_8	1.09 (0.55, 2.17)	0.802
Age	3.18 (1.44, 7.06)	0.004
Women (ref.: men)	0.52 (0.14, 1.89)	0.318
CHD mortality^b^		
amax21rrcor	1.20 (0.47, 3.07)	0.700
QTVInu	1.00 (0.59, 1.70)	0.990
SPPA_r_8	1.15 (0.38, 3.47)	0.801
Age	3.20 (1.04, 9.80)	0.042
Women (ref.: men)	1.59 (0.22, 11.33)	0.645
Triglycerides	0.96 (0.27, 3.42)	0.949
Type 2 diabetes subsample		
Variable selection dataset (*N* = 185; *n* = 62^a^, 32^b^)	Model fitting dataset (*N* = 47; *n* = 14^a^, 8^b^)	
CVD mortality^a^		
	HR (95% CI)	*P*
Age	2.05 (1.13, 3.75)	0.019
CHD mortality^b^		
	*HR (95% CI)*	*P*
a31rr	0.60 (0.32, 1.12)	0.109
ASC	0.64 (0.26, 1.58)	0.328
Age	1.67 (0.74, 3.78)	0.216
Women (ref.: men)	0.35 (0.06, 2.15)	0.259
Educational attainment	0.82 (0.26, 2.59)	0.731

amax21rrcor, slope of the auto-correlation function (acor) from the maximum of acor to the greatest auxiliary maximum of acor; QTVInu, QT Variability Index’s numerator multiplied by 1000; SPPA_c_8 ; number of data points in the 8th column of a 12 × 12 matrix; SPPA_r_8, number of data points in the 8th row of a 12 × 12 matrix; a31rr, slope of the auto-transformation function (ati) from the maximum of ati (tau = 0) to the subsequent value (tau = 2); ASC, portion of words of the pattern family “ascending” (the three successive symbols describe a ascending ramp (e.g. 2 3 4 or 0 1 2); HR, hazard ratio; CI, confidence interval; *P*, p-value; N, sample size; n, number of events; CVD, cardiovascular disease; CHD, coronary heart disease; HR is per one-SD increase for continuous variables; *adjusted HR.

### Bias analysis

RHR showed associations with CVD and CHD mortality risk in the crude model of the overall study sample and with CHD mortality risk in the prediabetes subsample; as such we evaluated the impact of follow-up. These associations were restricted to early follow-up suggesting the influence of follow-up time (Table S5). In general, HRs were considerably lower in late follow-up as compared to early follow-up. Relatedly, late follow-ups of RHR were inconsistent with entire follow-ups (Table S5). Further, Table S6 shows early and late follow-up associations of pNN50 with CVD mortality and CHD mortality in the overall sample, VLF and TP with CVD mortality in the NGT subsample, SDANN with CVD mortality in the prediabetes subsample, and SDANN and pNN50 with CVD mortality in the T2D subsample and SDANN with CHD mortality in the T2D subsample. The aforementioned associations in the overall sample and T2D subsample were only significant in late follow-up, whereas they were significant only in early follow-up in the NGT subsample. The association in early and late follow-up was non-significant in the prediabetes subsample. These results suggest an impact of follow-up. Additionally, the late follow-up results in the overall sample and the T2D subsample were similar to the entire follow-up, suggesting unlikely influence of reverse causality. On the other hand, late follow-up results in NGT and prediabetes being at variance with the entire follow-up suggest an influence of reverse causality. Besides, most HRs for the early follow-up were below one, but above one for the late follow-up.

The association of baseline, follow-up and change in RHR with CVD and CHD mortality risk (Table S7) as well as the association of baseline, follow-up and change in HRV indices with CVD and CHD mortality risk (Table S8) did not reveal any significant associations. The association of SDNN and RMSSD with CVD mortality was consistent between sexes (*P*_interaction_ > 0.05).

### Supplementary analysis

When dichotomizing HRV indices at the 5th percentile, several indices showed a higher association with CVD and CHD mortality for low HRV with the exception of low pNN50 that was associated with lower CVD mortality in the prediabetes subsample (Table S9). Further, RHR was associated with higher all-cause mortality in the overall sample (Table S10). Higher RMSSD, pNN50, VLF, LF and TP were associated with higher all-cause mortality in the overall sample and higher values of all indices except SDANN were associated with higher all-cause mortality in the T2D subsample (Table S11). Comparisons of continuous and spline models indicate that continuous models of RHR and indices generally outperform spline models (Table S12). The spline plots of RHR and indices for all-cause mortality in the overall sample and subsamples are displayed in Figures S11–S15. In the overall sample, all-cause mortality was higher in individuals <5th percentile of SDNN and RMSSD (Table S13), while in the T2D subsample, all-cause mortality was higher in individuals with individuals <5th percentile of SDNN, RMSSD and TP (Table S13). The analysis of the 66 indices and covariates showed that none of the recovered indices was validated for all-cause mortality (Table S14).

RHR showed low (|*r*|>=0.0-0.3) to moderate (|*r*|>=0.3–0.5) inverse correlations with most indices. Low correlations included associations with coefficient of variation of all NN intervals (cvNN) and ratio of LF and HF (LF/HF) and moderate correlations included associations with RMSSD (Table S15). Further, RHR was lower in individuals with prevalent myocardial infarction (Table S16). SDNN, Shannon_h and Rényi4 correlated strongly (|*r*|>=0.7) with most indices (Table S13). Covariates generally showed low correlations with indices (Table S15). Six indices including RMSSD, pNN50 and QT variability index were associated with prevalent myocardial infarction. They were higher in individuals with prevalent myocardial infarction (Table S16). All Supplemental materials are accessible at https://figshare.com/s/3f491c826e267b0c28f7.

## Discussion

The present study examined the overall and glucose tolerance status-specific associations of RHR and 66 HRV indices with CVD and CHD mortality among 1390 individuals from the population-based KORA cohort, who were aged 55 to 74 years at baseline and followed up for up to 22 years. After accounting for several covariates including age, sex, SBP, LDL-c, smoking, HbA1c and use of medications such as beta blockers, higher RHR was associated with higher CVD mortality and CHD mortality in the overall study sample. VLF was directly associated with CVD mortality in NGT, RMSSD showed a non-linear, S-shaped relationship with CVD mortality in T2D, while SPPA_c_8 was inversely associated with CVD mortality in prediabetes.

Our study revealed an association between RHR and CHD mortality risk, which has not been previously reported. Our observed association between RHR and CVD mortality risk is in line with the overall conclusion of a systematic review^4^ and a recent study showing that higher RHR is associated with higher CVD mortality risk^14^. Higher RHR was generally associated with higher CVD mortality risk^4^, albeit with inconsistencies in more recent studies^11–14^. Higher resting heart rate reflects the sympatho-vagal imbalance, essentially towards sympathetic predominance^4,52^. This suggests that dysfunctional autonomic nervous system activity is associated with higher risk of long-term CVD and CHD mortality. Of note, our bias analysis showed stronger associations for shorter (< 5 years) than for longer (> 5 years) follow-up times, especially in the total study sample and the subgroups with NGT and prediabetes. This indicates that baseline RHR measurements may better reflect the current physiological state including subclinical cardiovascular disease and therefore short-term CVD and CHD mortality risk than long-term cardiovascular trajectories and mortality risk beyond five years. It can be hypothesized that changes in other factors, such as increasing age, hypertension, deterioration of glucose regulation, dyslipidaemia or renal impairment, may increase in their importance as mortality determinants, while the predictive value of single RHR measurements decreases over time.

Further, VLF was directly associated with CVD mortality in individuals with NGT. VLF is one of the indices reflecting variability from mixed sources including sympathetic, parasympathetic, thermoregulation and functional capacity^15^. It is possible that this positive association in NGT represents a chance finding based on multiple tests, as there is as yet no biological explanation for such a difference compared to people with impaired glucose metabolism. There was a non-linear relationship between RMSSD and CVD mortality in individuals with T2D, in that there CVD mortality was lower for low and very high high values of RMMSD. For intermediate RMSSD, there was a positive association between RMMSD and CVD mortality. Others have reported a non-linear association of RMSSD with CVD mortality in 3136 women with T2D followed up for 9.7 years^31^, an association of RMMSD with CVD mortality in individuals with T2D^29^ and a combination of low RMSSD and low SDNN associated with CVD mortality risk in individuals with T2D following standard glycemic treatment^53^. Unlike Zhou et al.^31^ our finding is not modified by sex. RMSSD reflects the beat-to-beat variance in heart rate and it is the primary time-domain index used to estimate the vagally mediated changes in HRV^54^. In fact, five-minute RMSSD is considered the best marker of autonomic nervous system function^15^. It can be hypothesized that people at the higher end of RMSSD are characterized by a stronger vagal tone, better cardiovascular fitness and lower physiological stress, so that lower CVD mortality risk would be possible.

Finally, SPPA_c_8 was inversely associated with CVD mortality in individuals with prediabetes. This is one of the indices from the segmented Poincaré plot^55^. Our findings would be in line with SPPA_c_8 reflecting a stronger parasympathetic influence, so that it could be interpreted as a marker (rather than a cause) of healthier autonomic nervous system function. In contrast to the traditional Poincaré plot analysis (PPA), the SPPA retains the nonlinear features within beat-to-beat interval time series^56^. Because Column 8 sits near the central peak of the point distribution cloud, SPPA_c_8 accounts for a high percentage of overall beats (~ 13% in healthy populations). It reflects the stability of the neurocardiac baseline, i.e. how reliably the autonomic nervous system maintains normal, steady-state long-term oscillations rather than scattering into extreme, fragmented states. It has been suggested that SPPA may provide better risk stratification in conditions such as dilated cardiomyopathy when compared to traditional PPA^56^. Of note, only 5-minute ECG recordings are required, which are clinically feasible, but the complex statistical analyses for the calculation of SPPA_c_8 most likely exceed what is currently possible in routine clinical settings. The fact that the association of this index with CVD mortality is independent of multiple other indices and covariates in our study is intriguing but again, the potential pathophysiological and clinical relevance for CVD mortality in this particular subgroup remains elusive without corroborating studies.

Furthermore, we were able to confirm the absence of association of SDNN, LF and HF with CVD mortality in 159 individuals with T2D followed up for 9 years^32^ and absence of association of SDNN and RMSSD with CVD mortality in 4730 men with T2D followed up for 9.7 years^31^. The comparable age range of these studies, 50 to 75 years^32^, 40 to 79 years^31^ and ours, 55 to 74 years might be an explanation for the similar findings. Our findings of an absence of associations of SDNN, SDANN, VLF and LF with CVD mortality in the overall study is in contrast to the association observed in pooled analyses of other studies^15,16^. However, our study confirmed the absence of associations of HF and TP with CVD mortality reported in a pooled analysis of original studies^15^ as well as the absence of an association of TP with CVD mortality in a 7-year follow up in this cohort^37^. The generally direct relationship between continuously modeled indices and CVD mortality seems to be at odds with the general understanding of low HRV being detrimental to health. Nevertheless, the early follow-up of our continuous model (Table S6) generally showed the expected inverse relationship. Hence, it is likely that the long follow-up of our study and its associated relatively high proportion of events may have had an impact on the unexpected findings for the entire follow-up. As mentioned above for RHR, it appears possible that also baseline HRV variables are more reliable markers of baseline health and short-term risk than of long-term risk. The low proportion of events might explain the expected findings in the dichotomized analysis. Moreover, we found no association of 14-year change in RHR and HRV indices with CVD and CHD mortality in a subset of our study participants (*N* = 328). This finding is in line with a report of absence of association of five-year change in RHR in 8602 individuals and five-minute HRV indices in 3028 individuals with either fatal or nonfatal CVD^57^. Of note, non-linear HRV indices reveal complexities in heart rate patterns that cannot be perceived from linear indices^58^. Previous studies have shown associations between some non-linear indices and CVD mortality^17–20,33,34^. We were unable to confirm these previous findings either in the overall study population or in any of the glucose tolerance status subgroups. However, the null findings in our study should be seen in the context of our sample size that, especially in the subsamples, limited statistical power and entailed the risk of type II errors. Importantly, higher age was validated as an independent predictor of CVD mortality and CHD mortality in the overall study population and in individuals with NGT, which serves as a positive exposure control to demonstrate that our analytical approach could have uncovered additional associations for other predictor variables within the aforementioned limitations in statistical power.

One potential explanation for the association of only one HRV index, RMSSD, with CVD mortality in our study could be that impaired cardiac autonomic function, as captured by the current set of indices, is generally weakly associated with both non-fatal and fatal CVD events in our cohort. Actually, supplementary analysis of the association between indices and prevalent myocardial infarction confirmed this assertion, in that only six out of the 66 indices were associated with myocardial infarction. This is concurrent with a study in 342 individuals showing no association between HRV indices and non-fatal CVD events^19^. Thus, it is plausible that fewer indices will be associated with fatal CVD over a long period in our cohort.

Furthermore, in supplementary analyses, we observed associations between RHR and all-cause mortality risk in the overall study, but no reliable associations across glucose tolerance status groups, possibly related to the lower sample sizes in the subsets. This lends support to greater mortality risk in participants with high baseline RHR in a larger study^57^. Similar to CVD and CHD mortality, long follow-up of our study and the associated relatively high event to censored ratio could be the explanation of the positive HR that we observed for the association between continuously modeled indices and all-cause mortality. Although our continuously modeled RHR and RMSSD were superior to the non-linear model, however the non-linear relationships of RHR and RMSSD with all-cause mortality in our overall study sample are similar to those in another study^58^. When analyzing associations of HRV indices based on dichotomization at the 5th percentile, CVD and CHD mortality was generally higher for low values of several HRV indices with one exception. This approach can be useful of assessing the mortality risk of those with the most diminished HRV but ignores the rest of the distribution, so that additional analyses using non-linear plots are also informative. Indeed, an inspection of the non-linear plots of RMMSD in the overall study sample (Figures S11) and RMMSD and TP in individuals with T2D (Figure S14) indicates that mortality risk varies highly along segments of the indices. Dichotomized indices might obscure variabilities within the normal HRV range that could lead to a deeper understanding of the relationship between some HRV indices and mortality risk. Studies reporting associations only for categorized HRV indices could improve reliability of findings by validating the categories and showing that the relationship between indices and mortality risk are discontinuous at their cut-off points and flat between them.

Of note, it needs to be emphasized that the interpretation of associations between HRV measures and health ourcomes is complicated by the fact that high values for some measures may reflect a more erratic or disorganized system rather than better health in a subset of people^59^. Such erratic rhythms are also associated with higher age and higher CV risk. Given the general lack of significant associations between HRV indices and our outcomes we can at least largely exclude false-positive findings due to erratic rhythm. However, it is not possible to reliably assess to what extent this has led to false-negative findings.

In addition, our correlation analysis provides the magnitude of dependencies between RHR and HRV. The fact that RHR showed a moderate correlation with RMSSD suggests that each parameter covers partly distinct underlying mechanisms of CVD mortality. Moreover, the low correlation of RHR with other indices, particularly cvNN and LF/HF confirms the lower dependence of these indices on RHR^8^. We did confirm the well-known inverse association of RHR with HRV indices^8^. Some non-linear indices, such as portion of words of the pattern family “2LV” (ST_2LV) and slope of the auto-correlation function (acor) from the maximum of acor to the greatest auxiliary maximum of acor (amax21rrcor) have been linked to age and sex^55^. We confirmed these associations and observed novel findings for these indices, such as the association between ST_2LV and HbA1c as well as the association between amax21rrcor and BMI.

There are some limitations of our study. This is an observational study; as such, we cannot draw causal conclusions. We adjusted for multiple covariates, but residual confounding due to unmeasured factors related to RHR and HRV and advanced stages of various diseases that affect CVD survival, is possible. Unmeasured factors potentially associated with both HRV and CVD/CVD outcomes include, for example, past traumatic experiences that can have lasting effects on the autonomic nervous system and stress-response systems. Unfortunately, we did not have data on psychosocial trauma, chronic stress and other related conditions that could have been used in our analyses. It is, however, unlikely that any unmeasured covariate would be uncorrelated with any variable in our adjustment set, which would substantially alter the current conclusion. Indeed, additional adjustment for glucose tolerance status in the overall study sample did not affect the significant association of RHR with CVD and CHD mortality. Our indices were obtained from single short-term (5-minute) measurement at baseline. Findings from single long-term (24-hour) measurement may be different. While our findings appear less robust to intra-individual variation through analysis of one follow-up measurement and change in RHR and HRV, follow-up measurement within a shorter time from baseline such as less five years or more than one follow-up measurement at equally spaced intervals, might provide different conclusions. Moreover, most of our Cox models did not meet the rule of thumb of 10–20 events per predictor variable. However, our conclusion of absence of association still holds because adjustment for a few covariates, age, sex and SBP in the common indices analysis resulted in a similar conclusion. In addition, our glucose tolerance status-specific analysis had low to modest statistical power. Hence, their findings should be interpreted with caution because of potential type II errors. The finding that RHR and VLF associations attenuated with longer follow-up suggests baseline measures may partly reflect subclinical diseases rather than long-term risk.

Our study has several strengths. It is a prospective study with a relatively large sample size. The prospective design ensures temporal associations of RHR and HRV with mortality risk. It is one of the few studies on this topic with follow-up times of over 20 years. The long follow-up also ensured that we could assess reverse causation by excluding cases within the five years of follow-up. However, our reverse causality analysis suggests that the association of RHR and some HRV with CVD or CHD mortality risk might have been influenced by subclinical disease affecting mortality risk. Besides, specifically investigating CHD mortality helps to determine potentially unique findings for this heart disease, which is less reported in the literature. In addition, flexible modeling of non-linear associations by splines helps identify associations that would otherwise have been missed using continuous or ad-hoc categorical indices. In addition, multivariable analysis of indices with mortality allows crucially important indices to be uncovered within their complex interactions, while avoiding the shortcomings of univariable analysis. Finally, we accounted for important covariates such as HbA1c, hsCRP as general biomarker of subclinical inflammation, and medications, which previous studies rarely considered.

In conclusion, this prospective study showed that RHR indicating baseline autonomic tone is related to CVD and CHD mortality risk, while RMSSD, an HRV index providing insight into the autonomic nervous system-mediated modulation of the heart rate, influences CVD mortality risk in individuals with T2D. These findings suggest that RHR and HRV may provide differential and complementary insights into CVD mortality risk. These findings should be confirmed in independent population-based studies.

## Supplementary Information

Below is the link to the electronic supplementary material.

Supplementary Material 1

Supplementary Material 2

Supplementary Material 3

Supplementary Material 4

Supplementary Material 5

Supplementary Material 6

Supplementary Material 7

Supplementary Material 8

Supplementary Material 9

Supplementary Material 10

Supplementary Material 11

Supplementary Material 12

Supplementary Material 13

Supplementary Material 14

Supplementary Material 15

Supplementary Material 16

Supplementary Material 17

Supplementary Material 18

Supplementary Material 19

Supplementary Material 20

Supplementary Material 21

Supplementary Material 22

Supplementary Material 23

Supplementary Material 24

Supplementary Material 25

## Data Availability

The datasets generated and/or analyzed during the current study are not publicly available due KORA study’s restricted data use and access, but are available upon request, pending application and approval by the KORA study. To obtain permission to use KORA data under the terms of a project agreement, please use the digital tool KORA.PASST (https://helmholtz-muenchen.managed-otrs.com/external/).

## Abbreviations

a31rr: Slope of the auto-transformation function (ati) from the maximum of ati (tau = 0) to the subsequent value (tau = 2)
amax21rrcor: Slope of the auto-correlation function (acor) from the maximum of acor to the greatest auxiliary maximum of acor
BMI: Body mass index
CHD: Coronary heart disease
CI: Confidence interval
CRP: C-reactive protein
CVD: Cardiovascular diseases
cvNN: Coefficient of variation of all NN intervals
DBP: Diastolic blood pressure
ECG: Electrocardiogram
HbA1c: Glycated hemoglobin A1c
HDL-cholesterol: High-density lipoprotein cholesterol
HF: High frequency power
HR: Hazard ratio
HRV: Heart rate variability
ICD: International classification of diseases
KORA: Cooperative Health Research in the Region of Augsburg
LDL-c: Low-density lipoprotein cholesterol
LF: Low frequency power
LF/HF: Ratio of LF and HF
NGT: Normal glucose tolerance
NN: Normal-to-normal
OGTT: Oral glucose tolerance test
*P*: p-value
PH: Proportional hazards
pNN50: Percentage of the number of pairs of successive NN intervals differing by more than 50 ms
RMSSD: Square root of the mean squared differences of successive NN intervals
SBP: Systolic blood pressure
SDANN: Standard deviation of the averages of NN intervals
SDNN: Standard deviation of all NN intervals
SPPA_c_8: Number of data points in the 8th column of a 12 × 12 matrix
SPPA_entropy: Shannon entropy of all data points
SPPA_r_8: Number of data points in the 8th row of a 12 × 12 matrix
ST_2LV: Portion of words of the pattern family “2LV” (the three symbols formed an ascending or descending ramp (e.g. 1 2 4 or 5 4 3)
T2D: Type 2 diabetes
TP: Total power
VLF: Very-low frequency power
